# Improvement of glucose and lipid profile status with *Aloe vera* in pre-diabetic subjects: a randomized controlled-trial

**DOI:** 10.1186/s40200-015-0137-2

**Published:** 2015-04-09

**Authors:** Samaneh Alinejad-Mofrad, Mohsen Foadoddini, Seyed Alireza Saadatjoo, Majid Shayesteh

**Affiliations:** Faculty of nursing and midwifery, Birjand University of Medical Sciences, Birjand, Iran; Atherosclerosis and Coronary Artery Research Center, Birjand University of Medical Sciences, Birjand, Iran; Deputy of health, Birjand University of Medical Sciences, Birjand, Iran

**Keywords:** Aloe vera, Pre-diabetis, Fast blood glucose, Lipid profile, Herbal medicine

## Abstract

**Background:**

Pre-diabetes is a disturbing trend in the population, who are at risk of developing type-two diabetes. The aim of this study was to determine the effects use of Aloe vera in different doses on glucose and lipid profile in pre-diabetic subjects.

**Methods:**

This study was a double blind randomized controlled trial (72 subjects) with pre-diabetes symptoms in 3 groups consumed capsules twice a day: Aloe vera 300 mg (AL300), 500 mg (AL500) and placebo (PL). Fasting blood glucose (FBS), HbA1C and lipid profile were evaluated in baseline, 4 or 8 weeks. On-way ANOVA, Friedman, Wilcoxon, Kruskal-Wallis , Mann–Whitney and Chi-square tests were used for within or between groups statistical analysis.

**Results:**

FBS level in group AL300, showed significantly decreased in fourth week after the intervention, compared to PL in the same time (p = 0.001). Also, HbA1C level in this group at the eighth week after the intervention (p = 0.042), had a significant decrease. The levels of Total cholesterol and LDL-C, only in the group AL500 (p < 0.001 and p = 0.01), was significantly reduced, along with HDL-C level improvement just after eight weeks (p = 0.004). Triglyceride level showed a significant decrease (p < 0.045) just after four weeks use of AL500.

**Conclusions:**

The Use of Aloe vera extract in pre-diabetic patients, could revert impaired blood glucose within four weeks, but after eight weeks could alleviate their abnormal lipid profile.

## Introduction

Pre-diabetes and metabolic syndrome, which follows it, are parts of interrelated common clinical disorders that are accompanied by symptoms of obesity, insulin resistance, glucose intolerance, lipid abnormalities, impaired fasting glucose, and Impaired glucose tolerance (IGT) [1]. According to statistics, 470 million people will be suffering pre-diabetes by 2030 [2]. Some studies have reported that approximately 5-10% of pre-diabetic population would suffer from diabetes and problems associated with it in around a year such as heart problems, imbalance in glucose and lipid metabolism, and vascular disorders. Timely interventions in this population can preserve pancreatic beta cells, and improve their performance [3]. Current medications to control blood glucose and lipid profile may have dangerous side effects over time such as increased risk of weight gain, liver toxicity, and cardiovascular diseases [4]. Thus, we need to use stronger alternatives with less side effects. In this line, nutritional interventions, change in lifestyle, and behavioral therapy are on the rise. However, these interventions may not be effective alone to prevent the development of type 2 diabetes [5].

Aloe (*Aloe vera* L., Liliaceae family) has applications in health and cosmetic products as well as antioxidant, anticancer, anti-inflammatory, laxative, anti-atherosclerosis properties. It includes 75 active components that contain vitamins, enzymes, minerals, sugars, Lignin, salicylic acid, and amino acids [6,7]. Plenty of major components such as: Aloe-emodin, Aloetic-acid, Anthranol, Barbaloin, Mannan and its derivatives, 8-C-glusoly-(2′-O-cinnamoly), −7-O-methlyaloediol A, Alkaline phosphatese, amylase, bradykinase, carboxypeptidase, catalase, cyclooxidase, cyclooxygenase, lipase, oxidase, phosphoenolpyruvate, carboxylase, superoxide dismutase, Calsium, Chlorine, Chromium, Copper, Iron, Magnesium, Arachidonic acid, Y-linolenic acid, steroids, Mannose, glucose, L-rhamnose, Aldopentose, Vitamin A, B12, C, E, choline and folic acid, Auxins and Gibberellins have been found [8].

In one study, the use of oral administration of *Aloe vera* leaf gel extract for 21 days improved glycoprotein metabolism in diabetic animal models [9]. There is also evidence which shows that the glucose metabolism can be regulated with *Aloe vera*. The plant has other properties such as the reduction of hepatic tissue damage resulting from diabetic complications in rats [10] and reduction of the oxidative damage in the hippocampus and cerebral cortex of mice with type 2 diabetes [11].

Although hypoglycemic properties of *Aloe vera* have been reported for years [12], few human studies have been carried out so far [7,13-15]. Most of these studies have been implemented on patients with diabetes while some of such studies suffered from methodological weaknesses.

Individuals at risk for type 2 diabetes are increasing. On the one hand, simple and available remedies are required; on the other hand, no side effects for *Aloe vera* gel extract have been reported [14,15]. Thus, the present study was conducted to investigate the effects of two different doses of *Aloe vera* extract, and to determine the least time needed, to improve glucose and lipid profile levels in pre-diabetes subjects.

## Materials and methods

This study was a double blind randomized controlled trial done in the Research Center of Birjand University of Medical Sciences (BUMS) in November 2013. It was approved by the Methodology and Ethics Committee of BUMS on May, 2, 2013 with the 92.02.03 code number. Further, the trial was registered in the Iranian Registry of Clinical Trials with the number IRCT2013041112984N1.

This study was done on 72 volunteers who were selected according to entry criteria including an age between 35 to 65 years; fasting blood glucose between 100 and 125 mg/dl; HbA1C between 5.7 to 6.4; TG between 200–150 mg/dl; CHOL-C between 250–200 mg/dl; HDL-C less than 35 mg/dl; LDL-C between 130–160 mg/dl; BMI between 25–30; no use of any lipid-lowering treatment and no has any serious stress during last two months. The exclusion criteria involved a serious stress in the last 8 weeks; a history of type 2 diabetes or gestational diabetes; pregnancy; breastfeeding; allergy to *Aloe vera* or products containing it before or during the study; dissatisfaction to continue the study; liver and kidney disease; chronic respiratory problems or thyroid problems or infections that are inexcusable; elective surgery in the last two months; and absence from study for more than one week.

At first, the methodology was explained to volunteers as clearly and simply as possible and then obtained their informed consent. We used a formula comparing difference between two means for independent groups (power calculation = 80%) based on Devaraj et al. [14] study results. According to purposive non-probability sampling, blocking randomization method was used for the random allocation of patients among three groups (n = 24): *Aloe vera* groups (AL300, AL500) and placebo group (PL). The AL300 and AL500 groups received two capsules per day which contained 300 and 500 mg pure powdered of *Aloe vera* extract (Barij Essence Company, Kashan, Iran). The PL group received capsules that contained micro-crystalline cellulose (Aldrich, America). All the participants took the capsules after breakfast and after dinner for as long as eight weeks.

At the beginning of the study, fasting blood sample was taken to check for glucose and lipid profiles through standard methods. Plasma variables were measured by chemistry analyzer (Prestige 24i, Japan) using biochemical kits (ParsAzmoon, Iran) in one laboratory. Their BMI was calculated (based on measured their height by Seca 206, Germany and their weight by Beurer-PS07, Germany) and their systolic and diastolic blood pressures (standard sphygmomanometer) were also measured by one nurse. Then, the package of capsules with the timeline of how to use them was given to the participants. Participants visited the Research Center to give blood sample at the end of the fourth and eighth weeks of the study. The researcher’s phone number was given to the participants for probable queries. In addition, all the participants were reminded to take the pills and were emphasized not to use any other herbal medicine through phone calls.

Finally, they were requested not to have any change in their diet and routine activities during the study.

### Statistical analysis

All results are presented as mean ± SD. Statistical analysis was done in SPSS (version 16, SPSS Inc, Chicaogo, IL) using one-way ANOVA followed by Tukey’s or Bonferroni tests for parametric variables. For non-parametric variables (HbA1C, CHOL-C, HDL-C), Friedman and Wilcoxon tests were used to analyze within-group changes, and Kruskal-Wallis and Mann–Whitney tests were used to assess changes between groups. Chi-square test was used for comparing sex status. Significance level was considered at p < 0.05.

## Results

It is a double blind randomized controlled trial that was done on 72 pre–diabetes volunteers. The mean age of participants in the study was 52.5 ± 0.8 years, and 70% of them were women. Demographic characteristics and studied parameters for the subjects in different groups are presented in Table 1.

**Table 1:** **Characteristics of participants in the three groups of the study**

**Variable**	**PL**	**AL300**	**AL500**	**P-value**
	**Number (Percent) or Mean ± SD**	**Number (Percent) or Mean ± SD**	**Number (Percent) or Mean ± SD**	
Age (years)	54.5 ± 7.7	49.5 ± 7.5	53.2 ± 9.9	0.14
Man	(6) 26.1%	(5) 21.7%	(10) 41.7%	0.29*
Woman	(17) 73.9%	(18) 78.3%	(14) 58.3%	
BMI (kg/m^2^ )	27.9 ± 1.2	28.6 ± 1	28.2 ± 1.1	0.14
Height (cm)	159.7 ± 4.5	159.2 ± 7.5	161 ± 7.5	0.6
Weight (kg)	71.4 ± 4.4	72.2 ± 7.4	74.4 ± 7.2	0.3
HbA1C	6 ± 0.16	6 ± 0.24	6 ± 0.23	0.37
CHOL-C (mg/dl)	242.7 ± 3.13	241.4 ± 6.9	234 ± 1	0.14
HDL-C (mg/dl)	31.9 ± 1.2	30.4 ± 1.5	31.1 ± 2	0.74
LDL-C (mg/dl)	149.7 ± 5.2	148.7 ± 4.5	156.1 ± 5.1	0.12
Triglyceride (mg/dl)	173 ± 6.7	176.4 ± 7.5	179.7 ± 1.1	0.07
FBS (mg/dl)	110.1 ± 3.9	112.2 ± 2.5	111 ± 4.1	0.149
Systolic blood pressure (mmHg)	127.8 ± 3.3	125.8 ± 4.1	126.2 ± 4.7	0.23
Diastolic blood pressure (mmHg)	53.4 ± 5.5	50.2 ± 4.6	51.8 ± 5	0.10

*Chi-square test was used to check the homogeneity of gender in the three groups; one-way analysis of variance was used to examine the homogeneity in other variables at baseline.

**Figure 1:**
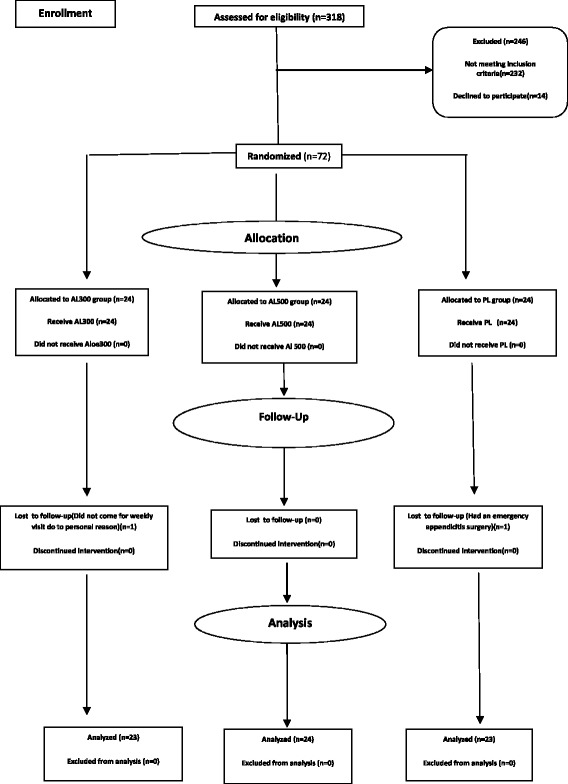
**Flowchart of the design, groups, and the participants in the project.**

The sample of the study included 72 volunteers. However, towards the end of the intervention, one participant from 300 mg capsule of *Aloe vera* left the study for unwillingness to continue the study, and one person from the placebo group had to leave for an emergency appendicitis operation. Finally, the sample involved 70 people (see Figure 1).

Chi-square and ANOVA test indicated homogeneity of demographic variables in different groups. Blood pressure of participants was not high at baseline, nor at the end of the intervention (Data not shown). No adverse effects were reported during the use of *Aloe vera* extract. Capsules were well tolerated and no complaint was received about the taste or smell of them. Usage of *Aloe vera* extract during the 8-weeks had the following results:

Fasting blood glucose levels in AL300 and AL500 groups decreased significantly four weeks (respectively: p = 0.006 and p = 0.001) and eight weeks (respectively p = 0.002, p < 0.001) after the intervention compared with those of the control group during the same time span. In fact, fasting blood glucose levels had no change in the control group during the study. Also, HbA1C level in both case groups had a significant decrease in the eighth week of the study (respectively, p = 0.042 and p = 0.011), compared with that of the control group (Table 2).

**Table 2:** **Comparison of the level of glucose and lipid profiles in** ***Aloe vera*** **- and placebo-treated groups**

**Variables**	**Time**	**PL**	**Within group**	**AL300**	**Within group**	**AL500**	**Within group**	**Between group**
		**N = 23**	**P-value**	**N = 23**	**P-value**	**N = 24**	**P-value**	**P-value**
**FBS (mg/dl)**	Before intervention	110 ± 3.91	-	112 ± 2.5	-	111 ± 4.1	-	0.69
	Fourth week	109 ± 4.14	0.92	109 ± 2.77*	0.006	108 ± 3.9*	0.001	0.009*
	Eighth week	110 ± 4.22	0.19	108 ± 2.78*	0.001	104 ± 4.2*	<0.001	0.001*
**Hb A1C (%)**	Before intervention	6.01 ± 0.16	-	6 ± 0.24	-	6 ± 0.23	-	0.37
	Eighth week	6.03 ± 0.14	0.059	5.8 ± 0.21*	0.042	5.6 ± 0.33*	0.011	0.04*
**LDL-C (mg/dl)**	Before intervention	149.7 ± 1.08	-	148.7 ± 0.94	-	151.6 ± 1.04	-	0.12
	Fourth week	150 ± 1.13	0.29	147.5 ± 0.52	0.24	146 ± 1.32	0.12	0.06
	Eighth week	150.7 ± 1.2	0.34	145.4 ± 1.31	0.37	137.5 ± 1.44*	0.01	0.01*
**CHOL-C (mg/dl)**	Before intervention	242 ± 3.13	-	241 ± 6.9	-	243 ± 10.4	-	0.21
	Fourth week	242 ± 3.24	0.73	238 ± 6.4	0.25	228 ± 11.6	0.053	0.04*
	Eighth week	243 ± 3.52	0.92	235 ± 8.1	0.13	218 ± 11.81*	<0.001	0.006*
**TG (mg/dl)**	Before intervention	173 ± 1.41	-	176.4 ± 1.5	-	179 ± 2.26	-	0.78
	Fourth week	173.3 ± 1.41	0.54	178.2 ± 2	0.19	174.8 ± 2.35	0.045	0.1
	Eighth week	173.6 ± 1.41	0.82	175.4 ± 2.08	0.072	168.6 ± 2.87*	0.005	0.03*
**HDL-C (mg/dl)**	Before intervention	31.95 ± 1.29	-	30.43 ± 1.50	-	31.1 ± 2.05	-	0.26
	Fourth week	31.82 ± 1.15	0.18	33.6 ± 3.99	0.25	33.6 ± 3.99	0.11	0.01*
	Eighth week	31.69 ± 1.45	0.09	35.3 ± 3.73*	0.08	35.3 ± 3.73*	0.004	0.007*

The within groups p values are for the comparison between the fourth week or the eighth week with baseline in the same group. * p <0.05: compared with the corresponding time in the placebo group.

All data are expressed as Mean ± SD. PL = placebo group. AL300 = the group that received 300 mg capsule of *Aloe vera*. AL500 = the group that received 500 mg capsule of *Aloe vera.*

Only in AL500 group, the amount of CHOL-C and LDL-C had a significant decrease in the eighth week (respectively, p < 0.001 and p = 0.01) while their HDL-C levels in the group increased (p = 0.004) at the end of the study. Furthermore, the results of this study indicate that consumption of four to eight weeks of the 500-mg capsules of *Aloe vera* can significantly reduce the levels of triglycerides of plasma (respectively p = 0.045 and p = 0.005) compared with baseline.

To compare the effects of different doses of *Aloe vera* between the groups, the mean changes of each parameter during similar time periods were calculated and analyzed. Levels of FBS and HbA1C in case groups had a significant decrease compared with corresponding periods in the control group. Levels of CHOL-C, LDL-C, and TG in just eight weeks after receiving 500 mg capsules of *Aloe vera* extract showed a significant decrease compared with the same values of the control group. However, regarding HDL-C level, both 300 and 500 mg doses of *Aloe vera* extract significantly reduced compared with the control group (p < 0.05) at the eighth week of the study.

## Discussion

Pre-diabetes is a serious global epidemic that increases the risk of getting type 2 diabetes by fivefold and the probability of cardiovascular disease by two times. A primary preventive measure for this disease is lifestyle modification, which is usually difficult. Therefore, it is necessary to utilize other therapies to reduce the consequences of this disease [16,17].

In the present study, the effects of different doses of *Aloe vera* extract at different time spans were studied on blood glucose and lipids in diabetic patients. The results of this study showed that both the 300 and 500 mg *Aloe vera* capsules within one and two months significantly reduced fasting blood glucose levels. Also, the capsules significantly decreased hemoglobin by the end of the study. Similar studies indicate that *Aloe vera* extract is effective in increasing insulin sensitivity, reducing fasting blood glucose, and decreasing the level of HbA1C in patients with pre-diabetes during eight weeks [14]. Several human studies have reported the anti-diabetic effects of *Aloe vera* extract [13,15]. In models of type I diabetes laboratory animals, it is shown that *Aloe vera* extract had a similar effect on blood glucose to that of glibenclamide [18]. Even in patients who did not respond to glibenclamide alone, consumption of *Aloe vera* extract for 2 weeks could reduce fast blood glucose [13]. Other studies have also shown the effectiveness of *Aloe vera* extract on the regulation of blood glucose levels in diabetic animals [9,19]. Few studies have indicated a rise in blood sugar levels after consumption of *Aloe vera* extract [20] which might be related to the use of different parts of the plant (not the gel) or short duration of the intervention (2 times a day for 3 days).

Researchers have also introduced an important element in the hypoglycaemic effects of *Aloe vera* in a substance called Acemannan. This is actually a D-isomer of compound polysaccharide that is extracted from *Aloe vera* leaf gel and has such properties as anti-virus, anti-cancer, digestive, and immune stimulating properties [14]. *Aloe vera* also contains other compounds such as hydrophilic fiber, glucomannan [21], and phytosterol [22] that reduce blood glucose.

It is suggested that *Aloe vera* can increase insulin sensitivity in the cells with reduce the level of blood glucose and insulin in serum [19] perhaps the *Aloe vera* can increase the Insulin Genetics activity In pancreatic beta cells. Study on the effects of anti-diabetic extract of *Aloe vera* showed that this plant cannot reduce the level of blood glucose in non-diabetic animals which is contrary to the results for hypoglycemic effects of glybenclamid [23].

The results of this study showed that AL500 could significantly reduce the levels of total cholesterol, TG, and LDL-C, and increase the level of HDL-C significantly during the 60 days of drug intake. However, AL300 could only increase the level of HDL-C in 60 days than before. For the first time, Agrawal studied the effect of *Aloe vera* on 5000 patients with type 2 diabetes for 5 years. After this period, a significant reduction was shown in the level of total cholesterol and triglycerides [12] that is consistent with our data. In another study, *Aloe vera* extract in dose of 300 mg was given on a daily basis to patients with type 2 diabetes for two months. The results indicated a significant reduction in the level of total cholesterol and LDL-C that are similar to our results. However, *Aloe vera* did not affect the level of HDL-C and triglycerides [15]. The reason may lie with the fact that only the 300-mg capsules of *Aloe vera* were used in that study on a population of type 2 diabetic patients who had high levels of blood glucose where, we know, that high levels of blood glucose can cause complex problems such as stress oxidative that will lead to the development of type 2 diabetes [24].

Also, it has been demonstrated that acute and chronic increase in the levels of blood glucose could increase the level of serum lipids (cholesterol, triglycerides, LDL, VLDL and decreased levels of HDL) [25]. The researcher believes that the reason for lack of response to this dose of *Aloe vera* (300 mg) capsules in this study was high chronic level of blood glucose in patients or the low dose of *Aloe vera*. It is conjectured that *Aloe vera* can bring the distribution of fatty acids in the blood to normal status by controlling the metabolism of lipids in the liver. In fact, *Aloe vera* extract can construct non-saturated fatty acids that remove free radicals from blood stream and control the metabolism of lipids in the body [18].

It is known that beta Sistostrol, Camposterol, and Stigmosterol are of close similarity to Phytosterols. Besides, it is found that beta Sistostrols chain available in some plants such as *Aloe vera* can significantly decrease the level of plasma total cholesterol, LDL-C, and triglycerides by inhibiting activation of fat absorption mechanisms [26]. In one study, it was shown that the use of *Aloe vera* extract as much as 200 mg/kg on a daily basis for as long as 100 days can significantly reduce the level of cholesterol, Triglycerid, free fatty acids, and phospholipids in normal mice [27]. It is also shown that taking *Aloe vera* extract for 8 weeks in diabetic rats can lower the level of cholesterol and TG [19]. Nonetheless, *Aloe vera* is beneficial even in short-term intakes (21 days) of 300 mg dose [24]. Some studies have mentioned that maximal dose of 50 mg *Aloe vera* could not improve the level of cholesterol in diabetic rats [22]. This suggests that the dose of *Aloe vera* that is required to reduce the level of cholesterol of serum is higher than the dose needed for reducing the level of blood glucose.

In a clinical trial conducted on 36 patients with type 2 diabetes, it was found that *Aloe vera* could reduce the level of triglycerides but had no effects on the level of cholesterol after daily use of one tablespoon of *Aloe vera* along with glibenclamide for 6 weeks [13] which are not similar to the results of our study. Perhaps, high blood glucose which may in turn lead to increased levels of blood lipid in the patients may suggest that they were taking lipid-lowering drugs instead of complementary medicine. The patient took *Aloe vera* juice which was not of sufficient accuracy to determine the exact amount of medication received. Devaraj and colleagues also showed, that taking two 500 mg capsules of *Aloe vera* (AC952) on a daily basis was effective in lowering the level of LDL and total cholesterol, is consistent with the results of our study. However it was not found to be effective in reducing the level of triglyceride and increasing the level of HDL-C of the serum [14]. Perhaps, it was because of the small number of participants in each group (n =15) or the little amount of active ingredients in the gel of, which can be as a result of the specific method of pasteurization and separation of the extract.

It was also reported that a high intake of *Aloe vera* (2 tablespoons three times daily for 12 weeks) could reduce the level of triglyceride of serum without effect on the levels of cholesterol, while no renal or hepatic toxicity was observed [7]. Increased activity of hormone-sensitive lipase during insulin secretion defect, increased release of free fatty acids from fat tissue. Thus, it produces more phospholipids and cholesterol in the liver due to accumulation of fatty acids in plasma. These two substances are released into the bloodstream as triglycerides which can increase the level of lipoproteins in the blood. It is suggested that can reduce the level of lipids in blood by controlling the fat metabolism in the liver [28]. Another theory is that the *Aloe vera* extract can lower the level of blood glucose and lipid in diabetic rats by improving sensitivity of cells to insulin [19,29]. It is well known that *Aloe vera* extract can suppress the adipogenesis gene and suggested that the plant can improve insulin resistance by reducing toxic effects of fat in the liver [19].

We had some limitations in quantitative control of some confounder variables such as food intake which could be considered for future researches.

## Conclusion

Use of *Aloe vera* extract in pre–diabetic patients can significantly regulate levels of fast blood glucose within four weeks and revert the levels of lipid profile, within eight weeks. It could be an interesting supplement strategy to alleviate impaired serum glucose level and lipid profile.
